# Mitochondrial dysfunction as a therapeutic nexus in HFpEF: therapeutic target and pharmacological advances

**DOI:** 10.3389/fphar.2025.1676988

**Published:** 2025-09-29

**Authors:** Tian Yue, Dezhi Zheng, Jian He, Shiqiang Xiong, Junbo Xu, Jun Hou

**Affiliations:** ^1^ Department of Cardiology, The Third People’s Hospital of Chengdu/Affiliated Hospital of Southwest Jiaotong University, Chengdu Institute of Cardiovascular Disease, Chengdu, Sichuan, China; ^2^ Department of Cardiovascular Surgery, The 960th Hospital of the PLA Joint Logistic Support Force, Jinan, Shandong, China

**Keywords:** HFpEF, mitochondrial dysfunction, therapeutic strategies, mitochondrial targeting, drug development

## Abstract

Heart failure (HF) with preserved ejection fraction (HFpEF) accounts for approximately 50% of all HF cases, and its incidence continues to rise with population aging and the surge in metabolic diseases. Unlike heart failure with reduced ejection fraction (HFrEF), HFpEF lacks effective therapeutic regimens to improve prognosis, with a 5-year mortality rate as high as 50%. Mitochondrial dysfunction, as a key link connecting metabolic disorders and abnormal myocardial systolic and diastolic function, has become a critical mechanism in the pathophysiology of HFpEF and a potential therapeutic target. This review systematically elaborates on the molecular mechanisms in HFpEF, such as mitochondrial energy metabolism disorders, dynamic imbalance, oxidative stress injury, and calcium signal dysregulation, comprehensively reviews the evidence for the effects of marketed drugs and drugs in clinical trials that improve mitochondrial function, and simultaneously explores emerging therapeutic strategies targeting mitochondria. This review aims to provide a theoretical reference for mechanistic research and drug development of HFpEF and promote the application of precision therapy targeting mitochondrial dysfunction in clinical practice.

## 1 Introduction

Heart failure with preserved ejection fraction (HFpEF) is a complex clinical syndrome characterized primarily by elevated left ventricular filling pressure and impaired diastolic function. In contrast, the left ventricular ejection fraction (LVEF) is normal or near normal (≥50%) (Rao et al., 2025; Hamo et al., 2024). Epidemiological studies have shown that HFpEF accounts for approximately 50% of all heart failure hospitalizations (Hamatani et al., 2025), and its prevalence is increasing significantly, which is closely associated with global population aging, obesity, and the epidemic of metabolic diseases such as type 2 diabetes (Rao et al., 2025; Rambarat et al., 2025). Patients commonly present with comorbidities such as hypertension (Redfield and Borlaug, 2023), obesity (Kosiborod et al., 2024a), and chronic kidney disease (Kirkman et al., 2021), as well as drug-drug interactions resulting from polypharmacy. These two factors exacerbate diagnostic confusion (Cheng and Nayar, 2009). Compared with heart failure with reduced ejection fraction (HFrEF), HFpEF patients have more limited treatment options. Most neurohormonal antagonists, such as Sacubitril/Valsartan (Jackson et al., 2021), effective for HFrEF have failed to show significant benefits in HFpEF treatment, resulting in a 5-year mortality rate as high as 50%, posing a severe public health challenge (Hamatani et al., 2025).

The pathophysiological mechanisms of HFpEF are highly heterogeneous, involving multiple factors such as systemic inflammation (Panico et al., 2024; Thorp and Filipp, 2025), microvascular dysfunction (Grootaert et al., 2025), myocardial fibrosis (Yamada et al., 2025), and metabolic disorders (Kumar et al., 2019). Recent studies have revealed that cardiomyocyte mitochondrial dysfunction is a core link throughout the occurrence and development of HFpEF, especially occupying a central position in HFpEF subtypes related to metabolic abnormalities (Givvimani et al., 2012; Madreiter-Sokolowski et al., 2024). As the energy powerhouse of cells, mitochondrial function directly affects the energy supply of the heart. Abnormal mitochondrial metabolism impairs fatty acid oxidation (FAO) processes (Fang and Gustafsson Å, 2024; Fonseka et al., 2025), reduces glucose metabolism efficiency, promotes increased glycolysis (Sun et al., 2024), and causes the accumulation of abnormal FAO metabolites and glycolytic products (Lim and Khunti, 2024). Such abnormal metabolic processes lead to dysfunction of the mitochondrial electron transport chain and electron leakage, during which reactive oxygen species (ROS) are generated. The accumulation of ROS triggers cellular oxidative stress activation (Gibb et al., 2021; Liang et al., 2025; Lozhkin et al., 2022; Gallo et al., 2024).

The accumulation of FAO metabolites and glycolytic products induces inflammatory activation, promotes the accumulation of inflammasomes such as NLRP3, and upregulates the secretion of inflammatory factors (Duewell et al., 2010). Over the long term, this leads to ventricular remodeling and fibrosis, further reducing the heart’s pumping capacity in HF (Pepin et al., 2025). In HFpEF, imbalances in mitochondrial dynamics and metabolic abnormalities cause intracellular calcium homeostasis dysfunction, severely impair myocardial diastolic function, and exacerbate HF (Hohendanner and Bode, 2020; McKenzie and Duchen, 2016).

This review elaborates in detail on the multi-dimensional roles of mitochondria in HFpEF, the current application and development of clinical and preclinical drugs, as well as the research status of active components of traditional Chinese medicine in HFpEF. It aims to provide new insights into a comprehensive understanding of the critical role of mitochondria in HFpEF and the development of mitochondria-targeted drugs.

## 2 Mitochondrial function and clinical treatment in HFpEF

### 2.1 Mitochondrial dysfunction and energy metabolism dysregulation

**FIGURE 1:**
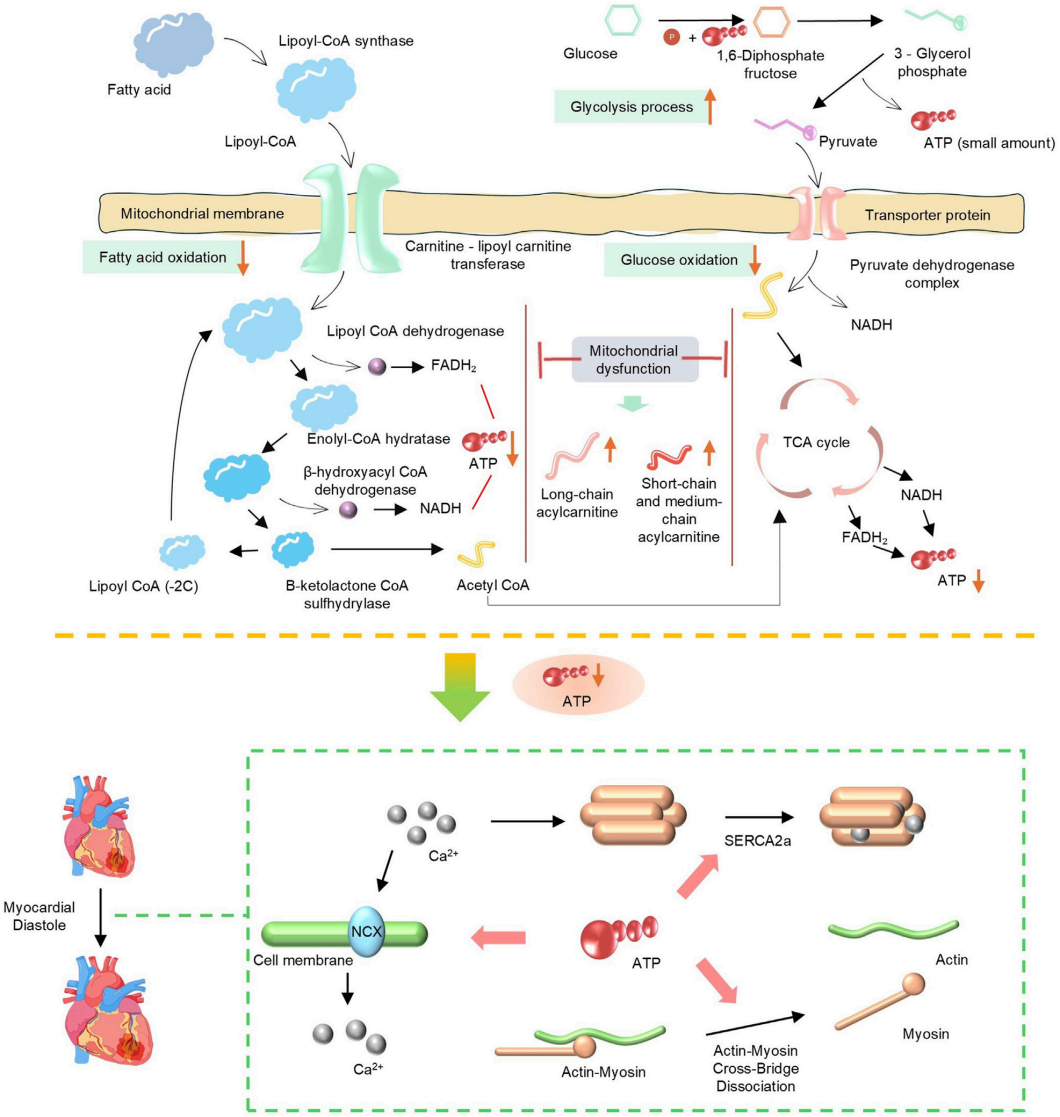
Schematic of mitochondrial energy metabolism pathways (fatty acid and glucose oxidation) and mitochondrial dysfunction, showing key enzymes, processes (TCA cycle, glycolysis), and impact on ATP production.

As a high-energy-consuming organ in the human body, more than 90% of the energy supply of cardiac muscle depends on mitochondrial oxidative phosphorylation (Figure 1) (Gallo et al., 2024; Shehadeh et al., 2025). In patients with HFpEF and animal models, cardiomyocytes exhibit a significant reduction in ATP synthesis, and the underlying cause lies in impaired utilization of energy substrates and impaired respiratory chain function (Shehadeh et al., 2025). FAO is a key pathway for cardiomyocytes to obtain energy (Mericskay et al., 2025). Under physiological conditions, the heart primarily utilizes fatty acids as an energy source, with more than 80% of ATP derived from FAO. FAO dysregulation is one of the core changes in mitochondrial energy metabolism in HFpEF, and it leads to further loss of cardiac function in HFpEF by affecting myocardial energy supply, stimulating the occurrence of oxidative stress, and aggravating ventricular remodeling (Zhang S. et al., 2025). AO dysregulation is typically associated with certain underlying metabolic diseases. For example, in HFpEF associated with obesity or diabetes, plasma levels of long-chain acylcarnitines are elevated, while short-chain and medium-chain acylcarnitines are increased in diabetic patients. Both indicate incomplete oxidation of fatty acids (FA), which largely limits mitochondrial ATP production (Hahn et al., 2023). Moreover, due to the abnormal accumulation of FA, increasing FA oxidation and the clearance rate of abnormally stored FA from tissues are therapeutically beneficial (Ying et al., 2021). Relevant studies have also confirmed this. In the HFpEF model induced by a high-fat diet in ApoE knockout (KO) mice, the levels of long-chain acylcarnitines in the myocardium are significantly elevated, and the proportion of saturated fatty acids increases abnormally, which directly promotes macrophage inflammatory response and cardiomyocyte dysfunction (Travers and Robinson, 2024). I addition, downregulation of peroxisome proliferator-activated receptor α (PPARα) signaling and decreased expression of carnitine palmitoyltransferase 1 (CPT1) further impair the efficiency of fatty acid β-oxidation. A recent study has shown that by modulating PPARα and preventing phosphorylation of nuclear factor κB (NF-κB), oxidative stress and inflammatory responses have been reduced (Zhou et al., 2024).

As another important energy source, abnormal glucose metabolism also significantly affects the progression of HFpEF. Against the backdrop of dominant fatty acid oxidation, the myocardium in HFpEF exhibits a “pseudohypoxic state”; even with normal coronary blood flow, the myocardium still tends to compensate for energy production through glycolysis (Mossmann et al., 2018; Watson et al., 2023). However, the ATP generated by glycolysis is far from sufficient to meet the energy demands of the myocardium, and HFpEF patients often have insulin resistance, resulting in reduced myocardial uptake and utilization of glucose (Hahn et al., 2023). yperglycemia and insulin resistance can stimulate the proliferation of cardiac fibroblasts, increase collagen synthesis, and lead to myocardial fibrosis. Meanwhile, abnormal glucose metabolism can also affect vascular endothelial function, reduce nitric oxide (NO) release, exacerbate coronary microcirculatory dysfunction and myocardial hypoperfusion, and further aggravate abnormalities in myocardial structure and function (Clyne, 2021). In addition, upregulated expression of PDH kinase 4 (PDK4) exacerbates phosphorylation and inactivation of pyruvate dehydrogenase (PDH), blocks the conversion of pyruvate to acetyl-CoA, and further impairs glucose oxidation capacity. A 2025 study revealed that HFpEF mice deficient in FGF21 exhibited significantly increased cardiac PDK4 expression, whereas FGF21 supplementation inhibited PDK4 by activating the PI3K/AKT pathway, restored PDH activity, and increased ATP synthesis (Piristine et al., 2024). Regardless of changes in FFA metabolism, HF is characterized by systemic neurohumoral activation and increased lipolysis, resulting in excess FFA supply that exceeds mitochondrial β-oxidation capacity, promotes the accumulation of toxic metabolic intermediates, and thereby leads to further mitochondrial dysfunction (Del Campo et al., 2021).

The mitochondrial electron transport chain drives transmembrane proton translocation through electron transport, forming a transmembrane proton gradient. Ultimately, Complex Ⅴ utilizes the gradient energy to synthesize ATP. Therefore, dysfunction of respiratory chain complexes is the direct cause of the energy crisis in HFpEF (Schauer et al., 2024). Respiratory chain damage leads to decreased efficiency of oxidative phosphorylation and reduced ATP production. When cardiomyocytes have insufficient energy reserves, the function of ATP-dependent diastolic calcium pumps, such as sarcoplasmic reticulum Ca^2+^-ATPase, is impaired, resulting in delayed clearance of cytoplasmic Ca^2+^ and impaired ventricular diastolic relaxation, which is one of the core mechanisms of diastolic dysfunction (Torre et al., 2022). For example, the ZSF1 rat HFpEF model shows that the activity of myocardial mitochondrial Complex I and III is significantly decreased, leading to impairment of electron transport and reduced ATP synthesis (Fopiano et al., 2024). Proteomic analysis further reveals that the stability of respiratory chain supercomplexes is decreased, particularly with reduced content of Cardiolipin, which impairs the assembly and function of the complexes. As a key phospholipid in the inner mitochondrial membrane, Cardiolipin is crucial for maintaining the spatial conformation of respiratory chain complexes and the efficiency of electron transport (Dolinsky et al., 2016).

Under normal physiological conditions, only 4%–15% of ATP in the myocardium is derived from ketone bodies, which is much lower than the contributions from fatty acids (40%–70%) and glucose (20%–30%) (Manolis et al., 2023). Ketone bodies originate from acetyl-CoA generated by fatty acid β-oxidation in hepatic mitochondria, and are converted into β-hydroxybutyrate, acetoacetate, and other ketone bodies through a series of enzymatic reactions (Manolis et al., 2023; Xu et al., 2023).

In terms of metabolic crosstalk, ketone bodies reduce glucose utilization by inhibiting phosphofructokinase (PFK), a key enzyme in glycolysis. Meanwhile, they indirectly regulate FAO through modulating carnitine CPT1, thereby preventing excessive lipid accumulation (Brahma et al., 2022). In heart failure with HFpEF, myocardial metabolic remodeling is accompanied by increased ketone body utilization, which serves as a compensatory mechanism. However, in the diabetic state, decreased activity of 3-oxoacid CoA transferase (SCOT) results in reduced ketone body uptake, exacerbating energy deficiency (Anker et al., 2021). In patients with chronic kidney disease (CKD), the renal capacity for ketone body clearance is impaired (renal clearance accounts for 28% under normal pH conditions), which may induce ketone body accumulation and oxidative stress. These abnormalities exacerbate HFpEF through mitochondrial dysfunction and reduced metabolic flexibility, whereas SGLT2 inhibitors may exert a protective effect by improving ketone body metabolism (Garcia-Ropero et al., 2019; Yurista et al., 2019; Solomon et al., 2022).

### 2.2 Imbalance in mitochondrial dynamics and dysregulation of quality control

**FIGURE 2:**
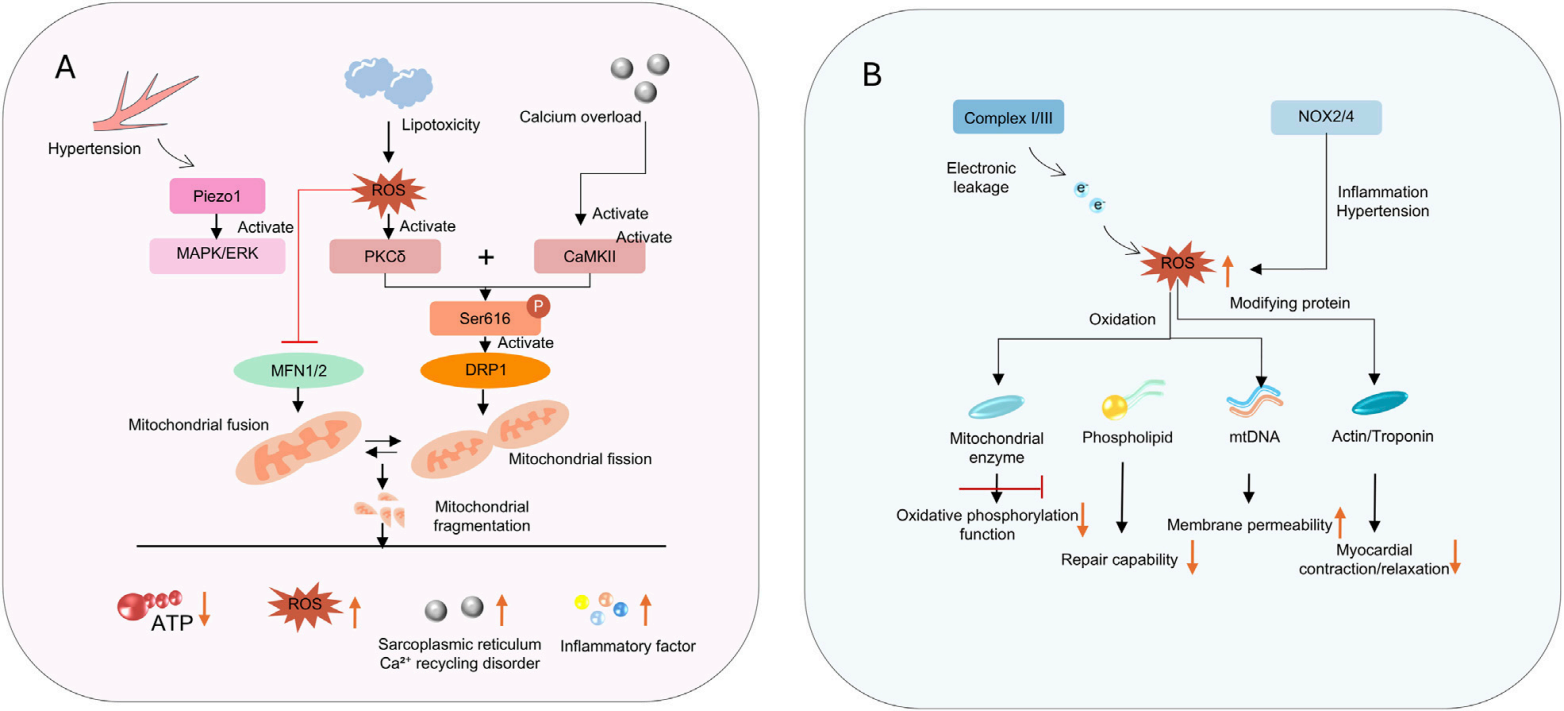
**(A)** shows signaling pathways disrupting mitochondrial dynamics; **(B)** depicts ROS - induced impairments in function and myocardial contraction.

Mitochondria maintain the dynamic balance of their network through continuous fission and fusion, and clear damaged components via mitophagy (Figure 2A). These processes are regulated by specific mitochondrial fusion and fission proteins, ensuring the integrity of mtDNA by eliminating mitochondria with damaged DNA and promoting functional units (Moral et al., 2020). In HFpEF, this dynamic balance is disrupted, characterized by excessive fission and impaired fusion. For example, a recent study has shown that abnormal mitochondrial content and decreased levels of MFN2 are observed in myocardial tissue of elderly HFpEF patients (Molina et al., 2016). In addition, stress factors such as a high-fat diet and hypertension can also induce the translocation of mitochondrial fission protein Drp1 to mitochondria, leading to mitochondrial fragmentation. Preclinical studies have confirmed that Drp1-mediated excessive fission not only reduces mitochondrial oxidative capacity but also promotes cardiomyocyte apoptosis. Conversely, downregulated expression of fusion proteins such as optic atrophy 1 (OPA1) and mitofusin 2 (MFN2) impairs mitochondrial network connectivity and compromises energy transfer efficiency. Some studies have shown that inhibiting Drp1 protein can alleviate left ventricular (LV) dysfunction in HFpEF associated with decreased LC3 and P62 (Givvimani et al., 2012). Furthermore, triggering PKA activation and inhibitory phosphorylation of Drp1 via miR-129-3p has been shown to exert cardioprotective effects (Sotomayor-Flores et al., 2020).

Mitophagy is an important process for the clearance of damaged mitochondria and mitochondrial renewal. Patients with HFpEF are usually accompanied by a decline in mitophagy function, failing to clear dysfunctional mitochondria and their accumulation in cardiomyocytes (Abdellatif et al., 2025a; Abdellatif et al., 2025b). On one hand, dysfunctional mitochondria cannot normally perform energy supply. On the other hand, they inhibit the function of normal mitochondria, further reducing energy supply in HFpEF, decreasing myocardial ATP reserves, affecting diastolic calcium ion homeostasis, and exacerbating myocardial diastolic dysfunction (Krammer et al., 2025). Accumulated dysfunctional mitochondria are one of the main sources of ROS release, which leads to cellular oxidative stress and inflammatory activation, and this part will be discussed in depth later (Yoshii et al., 2024). The PINK1/Parkin pathway is the main regulatory mechanism of mitophagy. In the context of HFpEF, chronic inflammation and oxidative stress disrupt the stability of PINK1, leading to insufficient clearance of damaged mitochondria and persistent accumulation of dysfunctional mitochondria. Studies have found that in obesity-related HFpEF models, mitophagy flux in cardiomyocytes is significantly reduced, which is negatively correlated with the degree of insulin resistance (Li et al., 2023; Qiu et al., 2019).

The Unfolded Protein Response (UPR) pathway serves as one of the core defense mechanisms for mitochondria to cope with protein misfolding stress and maintain their own homeostasis. By regulating the efficiency of protein folding in the mitochondrial matrix and promoting the degradation of abnormal proteins, it directly affects mitochondrial respiratory chain function and oxidative stress levels, and is crucial for maintaining the integrity of mitochondria in myocardial cells. Given the key role of the UPR pathway in regulating mitochondrial function, it has become an important therapeutic target for mitochondrial-related diseases. Currently, small-molecule modulators targeting this pathway (such as UPR activators) have entered the early-phase clinical trial stage, and preliminary effects of some drugs—including improving mitochondrial energy metabolism and alleviating myocardial cell damage—have been observed in animal models.

### 2.3 ROS injury and oxidative stress

The accumulation of ROS in HFpEF is caused by two factors (Figure 2B). First, there is increased ROS production (Nohara et al., 2019). Mitochondria are the main source of intracellular ROS, particularly when the electron transport chain is impaired. Increased electron leakage leads to excessive generation of superoxide anions (O2•−) (Delalat et al., 2025; Teuber et al., 2022). In addition to electron leakage from the respiratory chain, the expression of NADPH oxidase 4 (NOX4) is upregulated in cardiomyocyte membranes and mitochondria in HFpEF, directly promoting O2•− production (Teuber et al., 2022). Furthermore, lipid peroxidation products such as malondialdehyde (MDA) accumulate in myocardial tissue, reflecting the degree of oxidative damage (Costantino et al., 2023). Second, the function of the ROS scavenging system is reduced. The activity of mitochondrial antioxidant enzymes such as superoxide dismutase 2 (SOD2) and glutathione peroxidase 1 (GPX1) decreases, impairing ROS clearance capacity (Russomanno et al., 2017; Su et al., 2023).

The accumulation of ROS exacerbates cardiac dysfunction in HFpEF through multiple mechanisms (Jankauskas et al., 2021). ROS can directly act on the cytoskeleton of cardiomyocytes and affect the function of contractile proteins such as cardiac actin and myosin, impairing normal myocardial contraction and relaxation (Zuo et al., 2015). When acting on fibroblasts, ROS activate the transformation of fibroblasts into myofibroblasts, increase collagen deposition, and exacerbate myocardial fibrosis. Meanwhile, ROS can inhibit the synthesis and release of NO in vascular endothelial cells, impair vascular diastolic function, and increase cardiac load (Zuo et al., 2015). The accumulation of ROS and the state of oxidative stress in cardiomyocytes further damage mitochondrial function, leading to energy metabolism disorders and more ROS release, creating a continuous vicious cycle (Lozhkin et al., 2022).

### 2.4 Inflammation-metabolism crosstalk and microenvironmental dysregulation

**FIGURE 3:**
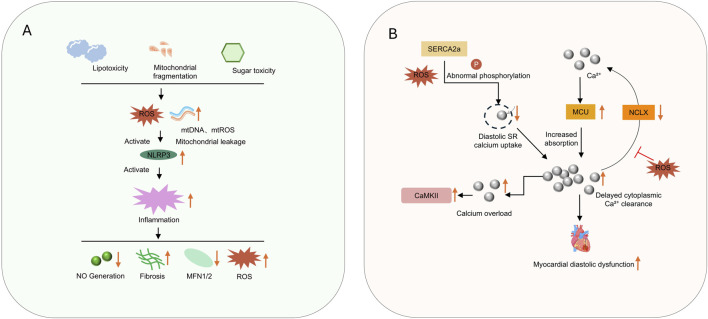
**(A)** shows lipotoxicity, mitochondrial fragmentation, and sugar toxicity triggering ROS mediated inflammation; **(B)** illustrates calcium dysregulation causing myocardial diastolic dysfunction.

The cardiac immune microenvironment and mitochondrial function form a tightly interconnected network, which is particularly prominent in metabolic HFpEF (Figure 3A) (Kosiborod et al., 2024b; Verma et al., 2024). Dysregulation of macrophage-cardiomyocyte crosstalk, macrophages are highly plastic and can differentiate into various phenotypes in response to microenvironmental signals (Panico et al., 2024; Deng et al., 2021). Studies have shown that in HFpEF hearts, the pro-inflammatory macrophage (M1) phenotype is significantly increased, while the proportion of TIM4+ macrophages, which possess anti-inflammatory and tissue repair functions, is decreased (Kopec et al., 2024; Zhang N. et al., 2022). Activated macrophages release pro-inflammatory factors such as interleukin-1β (IL-1β), tumor necrosis factor-α (TNF-α), and interleukin-6 (IL-6), which impair mitochondrial biogenesis by inhibiting the expression of PPARγ coactivator-1α (PGC-1α) (Agrawal et al., 2023; Gorica et al., 2025). Although anti-inflammatory phenotype macrophages (M2) can alleviate inflammation-induced damage to a certain extent, transforming growth factor-β (TGF-β) secreted by M2 macrophages is one of the potent profibrotic factors, which can activate the transformation of cardiac fibroblasts into myofibroblasts and promote the synthesis and deposition of collagen (types Ⅰ and Ⅲ) (Frangogiannis, 2024). Therefore, finding a balance in the phenotypic changes of different macrophages is crucial for HFpEF.

Furthermore, macrophages can inversely regulate the cardiac immune microenvironment. Matrix metalloproteinases (MMPs) released by macrophages can degrade the extracellular matrix (ECM) (Tourki et al., 2020). However, in HFpEF, the imbalance between MMPs and tissue inhibitors of metalloproteinases (TIMPs) leads to abnormal ECM remodeling and exacerbates fibrosis (Tourki et al., 2020; Krebber et al., 2020). Meanwhile, macrophages can further recruit more inflammatory cells and fibrosis-related cells by phagocytosing signaling molecules (such as ATP) released by apoptotic cardiomyocytes (Smart et al., 2023).

Accumulation of FA is caused by abnormal FAO, i.e., lipotoxicity, which also activates inflammation and exacerbates FAO abnormalities. Excess lipids accumulate as lipid droplets in cardiomyocytes, compressing organelles such as mitochondria and sarcoplasmic reticulum, disrupting the normal structure of cardiomyocytes, and reducing contractile-diastolic coordination (Janssen-Telders et al., 2025; Leggat et al., 2021).

Lipotoxicity is typically associated with obesity, metabolic syndrome, or type 2 diabetes (Fonseka et al., 2025). Incompletely oxidized lipid intermediates (e.g., ceramides, long-chain acyl-CoA, and lipopolysaccharides, LPS) can directly impair the integrity of cardiomyocyte membranes, inducing apoptosis (via activation of the caspase pathway) and necrosis (De Geest and Mishra, 2022; Ketema and Lopaschuk, 2025). For instance, in obesity-related HFpEF, elevated levels of cardiac local free FA (such as lauric acid and myristic acid) directly stimulate pro-inflammatory polarization of macrophages. *In vitro* experiments have confirmed that bone marrow-derived macrophages from ApoE knockout (KO) mice, when exposed to saturated fatty acids, show significantly upregulated expression of TNF-α and IL-6, forming a “metabolism-inflammation” vicious cycle (Phu et al., 2023; Korbecki and Bajdak-Rusinek, 2019; Vendrov et al., 2024; Riera-Borrull et al., 2017). Lipotoxicity also affects mitochondria: accumulated lipids (especially free fatty acids) in cardiomyocytes can enter mitochondria and undergo excessive oxidation, further impairing the function of the electron transport chain (Da Silva Rosa et al., 2021; Zhang J. et al., 2022).

### 2.5 Calcium homeostasis and mitochondria-sarcoplasmic reticulum coupling dysfunction

Under physiological conditions, calcium homeostasis in cardiomyocytes relies on the dynamic balance between calcium release during systole and calcium reuptake during diastole (Figure 3B) (Sutanto et al., 2020; Kho, 2023). Imbalance of calcium homeostasis directly impairs ventricular diastolic function and increases fibrosis, mainly manifested as diastolic calcium overload leading to increased diastolic tension in cardiomyocytes, slowed relaxation rate, elevated ventricular filling resistance, increased left ventricular end-diastolic pressure (LVEDP), and reduced compliance—classic hemodynamic features of HFpEF (Adeniran et al., 2015; Tu et al., 2025).

Sustained calcium overload can activate calcium-dependent proteases (e.g., calpains) and transcription factors (e.g., NFAT), promote cardiac fibroblast proliferation and collagen synthesis, exacerbate myocardial interstitial fibrosis, and further increase ventricular stiffness (Tu et al., 2025). By uptake of cytoplasmic calcium ions, mitochondria not only regulate calcium transient kinetics but also provide activation signals for calcium-dependent dehydrogenases (Mira Hernandez et al., 2025).

First, there is abnormal sarcoplasmic reticulum calcium handling: in HFpEF, the activity of sarcoplasmic reticulum Ca^2+^-ATPase (SERCA2a) is reduced, and phospholamban (PLB) is insufficiently phosphorylated, resulting in delayed diastolic calcium reuptake into the sarcoplasmic reticulum (Grote Beverborg et al., 2021). Excessive phosphorylation of ryanodine receptor 2 (RyR2) causes calcium leakage, reducing sarcoplasmic reticulum calcium storage (Dridi et al., 2023).

Second, there is dysregulated mitochondrial calcium uptake: dysfunction of the mitochondrial calcium uniporter (MCU) complex impairs mitochondrial calcium buffering capacity, leading to delayed cytoplasmic calcium clearance and prolonged myocardial diastolic duration (Weiser et al., 2023). Disrupted calcium signaling simultaneously reduces mitochondrial dehydrogenase activity, further inhibiting ATP production and forming a positive feedback loop between energy deficiency and abnormal calcium handling (Alves-Figueiredo et al., 2024).

### 2.6 Mitochondrial subtypes

In the myocardium of heart failure with preserved ejection fraction (HFpEF), there are functionally specialized mitochondrial subtypes, and their heterogeneous damage is a key link in disease progression (Glancy et al., 2017). Based on differences in spatial distribution and function, myocardial mitochondria can be divided into interfibrillar mitochondria (adjacent to myofibrils, responsible for immediate ATP supply) and perivascular mitochondria (near capillaries, involved in metabolic substrate exchange). Preclinical studies have shown that in the ZSF1-obese HFpEF rat model, the crista structure disruption, which directly leads to insufficient energy supply for the sarcoplasmic reticulum calcium pump (SERCA2a) and exacerbates diastolic dysfunction (Kumar et al., 2019).

Studies on human HFpEF samples further confirm that the proportion of aging-related mitochondrial subtypes (such as heteroplasmic mitochondria harboring mutant mtDNA) increases, which damages cardiomyocytes via oxidative stress (Tschöpe et al., 2017). These subtype-specific abnormalities suggest that targeted interventions for different mitochondrial subtypes (e.g., improving the crista structure of interfibrillar mitochondria, clearing abnormal perivascular mitochondria) may become a new direction for HFpEF treatment, addressing the previous lack of attention to functional differences among mitochondrial subtypes (Shou and Huo, 2022).

### 2.7 Clinical treatment of HFpEF

Currently, there is no consensus on the existence of curative drugs for HFpEF. Treatment is usually implemented from aspects including lifestyle modifications, management of underlying diseases, and symptomatic treatment (Heidenreich et al., 2022). Lifestyle is one of the key factors influencing the progression of HFpEF, mainly manifested in salt intake, weight changes, smoking, alcohol consumption, and so on. For HFpEF patients, daily salt intake should be strictly controlled below 5 g, while excessive fluid intake should be avoided to prevent edema caused by it McDonagh et al. (2021), Mullens et al. (2024). For patients with a body mass index (BMI) ≥30 kg/m^2^, it is necessary to lose weight, restrict the intake of high-calorie foods, and perform 150 min of moderate-intensity aerobic exercise per week. Additionally, tobacco and alcohol intake should be strictly restricted (Mullens et al., 2024).

Patients with HFpEF commonly have comorbid underlying diseases, such as hypertension, type 2 diabetes mellitus, and/or obesity and obstructive sleep apnea (Campbell et al., 2024; Gao et al., 2024). Therefore, strictly controlling these comorbid underlying conditions is one of the core priorities in the treatment of HFpEF. This includes the use of drugs that improve diastolic function, such as angiotensin-converting enzyme inhibitors (ACEIs)/angiotensin II receptor blockers (ARBs) or long-acting calcium channel blockers, to lower blood pressure (Wang et al., 2022). The use of SGLT2 inhibitors to control blood glucose and reduce the risk of heart failure hospitalization, and for patients with moderate to severe obstructive sleep apnea (OSA), the need for nocturnal continuous positive airway pressure (CPAP) therapy (Savarese et al., 2022). When patients present with increased volume overload, loop diuretics should be administered to reduce volume overload.

### 2.8 Inter-organ interaction and crosstalk

The development of HF with HFpEF is not only affected by cardiac function but also a consequence of the interaction of multiple organ dysfunctions. Pathologies of organs such as the lungs, liver, and kidneys can induce or exacerbate HFpEF through hemodynamic changes, neuroendocrine activation, and inflammatory responses (Shah et al., 2016; Borlaug et al., 2023). The heart and lungs are closely associated; certain pulmonary diseases, such as chronic obstructive pulmonary disease (COPD) (Abdo et al., 2025) and pulmonary embolism (PE) (Reddy et al., 2025), can increase pulmonary vascular resistance, thereby elevating cardiac afterload. Chronic hypoxia caused by COPD and PE activates the renin-angiotensin-aldosterone system (RAAS), leading to myocardial hypertrophy and further exacerbation of cardiac diastolic dysfunction (Mascolo et al., 2022). The kidney is one of the body’s main fluid balance-regulating organs, renal pathologies usually cause fluid retention and electrolyte disturbances, which directly induce or exacerbate HFpEF (Rovin et al., 2023). Electrolyte disturbances, such as changes in potassium and calcium ion levels, can also directly affect the systolic/diastolic function of the heart (Ranasinghe et al., 2024). The liver is one of the important organs regulating coagulation function and neuroendocrine hormones. Hypoalbuminemia resulting from reduced hepatic synthesis of albumin and coagulation factors directly promotes tissue edema and increases cardiac preload (Huynh, 2022). Therefore, HFpEF is a “vicious cycle network” formed by organs including the heart, lungs, kidneys, and liver through hemodynamic abnormalities, neuroendocrine activation (e.g., RAAS, sympathetic nervous system), chronic inflammatory responses, and metabolic disturbances.

## 3 Mitochondria-targeting therapeutic drugs

Protocols for improving HFpEF by regulating mitochondrial function mainly include direct targeting of mitochondria, regulation of mitochondrial energy metabolism, and acting on mitochondrial quality control (Table 1).

**TABLE 1:** Part of drugs acting on mitochondria for treating HFpEF and their mechanisms of action.

Category	Drugs/strategies	Mechanisms related to mitochondria	Citation
Direct mitochondrial targeting	SS-31 (elamipretide)	Binding Cardiolipin, stabilizing mitochondrial cristae, reducing MTROS, and improving electron transport	Russo et al. (2024)
	MitoQ	Mitochondria-targeted antioxidants, scavenging MTROS, improve electron transport	Ribeiro Junior et al. (2018)
Substrate and energy metabolism	Empagliflozin (SGLT2i)	Promote the utilization of ketone bodies, improve Na^+^/Ca^2+^ homeostasis, and indirectly improve mitochondrial homeostasis and autophagy	Lyu et al. (2023)
	Dapagliflozin (SGLT2i)	Metabolic remodeling to improve myocardial energy utilization	Li et al. (2025)
	Semaglutide (GLP-1 RA)	Weight loss, anti-inflammatory, metabolic improvement, myocardial GLP-1R is associated with mitochondrial signaling	Li et al. (2024a), Ma et al. (2024)
	Tirzepatide (GIP/GLP-1 RA)	Significant weight loss, metabolic improvement, indirect improvement of mitochondrial function	Malavazos et al. (2023)
NAD^+^/Mitochondrial quality control	Nicotinamide Riboside (NR)	Enhance NAD^+^, activate Sirtuins, promote mitochondrial biogenesis and quality control	Peng et al. (2024)
	Nicotinamide/NMN	Enhances NAD^+^ and promotes mitochondrial function	Peng et al. (2024)

### 3.1 SGLT2 inhibitors: remodeling mitochondrial metabolism and inflammatory balance

#### 3.1.1 SGLT2 inhibitors: mechanisms of action in the treatment of HFpEF

**FIGURE 4:**
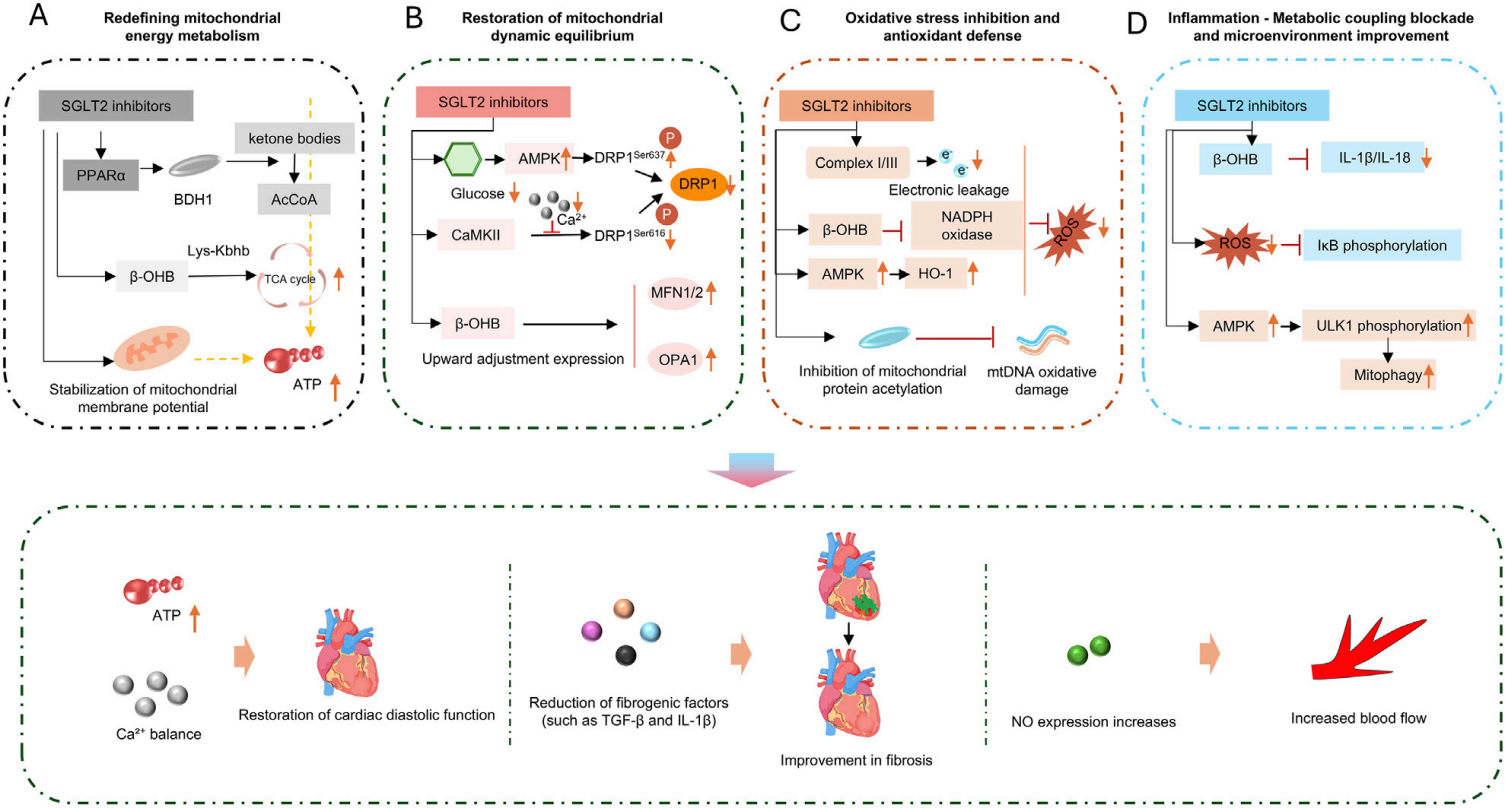
SGLT2 inhibitors act via four pathways: **(A)** energy metabolism; **(B)** dynamic equilibrium; **(C)** oxidative stress; **(D)** inflammation, to improve cardiac function, including restoring diastolic function and enhancing blood flow.

Sodium-glucose cotransporter 2 inhibitors (SGLT2i), initially developed as hypoglycemic agents, have been confirmed in recent years to exert a significant improvement effect on cardiac function and clinical outcomes in patients with HFpEF (Figure 4) (Frampton, 2022). Recent studies have demonstrated that SGLT2i exhibit great potential in restoring mitochondrial respiratory chain to improve energy metabolism, inhibiting inflammation, ameliorating oxidative stress, and alleviating myocardial fibrosis (Withaar et al., 2021; Zafeiropoulos et al., 2024).

The improvement of myocardial energy metabolism by SGLT2i is mainly reflected in its ability to reduce myocardial dependence on glucose while promoting ketone body metabolism (Goedeke et al., 2024). SGLT2i increase circulating ketone bodies, including β-hydroxybutyrate and acetoacetate, etc., providing cardiomyocytes with efficiently utilizable ketone bodies as an alternative energy source for ATP production. Unlike glucose, ketone body metabolism produces less NADH, which also reduces the pressure on the mitochondrial electron transport chain, decreases ROS production, and reduces cardiomyocyte dependence on glucose (Uchihashi et al., 2017).

In addition, SGLT2i also affects mitochondrial energy metabolism-related pathways. They may indirectly activate AMP-activated protein kinase (AMPK) by mildly reducing intracellular ATP levels, thereby upregulating peroxisome proliferator-activated receptor γ coactivator-1α (PGC-1α). As a core regulator of mitochondrial biogenesis, PGC-1α can promote the gene expression and protein synthesis of respiratory chain complexes (e.g., Complex Ⅳ) and enhance oxidative phosphorylation capacity (Hoong and Chua, 2021). For example, a recent study has shown that in the ZSF1 rat HFpEF model, Empagliflozin treatment for 8 weeks significantly improved the activity of myocardial mitochondrial Complex I and III and increased ATP production (Schauer et al., 2024). Mechanistic studies have indicated that this drug promotes cardiolipin remodeling by upregulating cardiolipin synthases (such as CRLS1 and TAZ), stabilizes the assembly of respiratory chain supercomplexes, and thus restores oxidative phosphorylation efficiency (Jhuo et al., 2023; Santos-Gallego et al., 2019). Proteomic analysis further revealed that Empagliflozin reversed 44% of HFpEF-related abnormal expression of mitochondrial proteins, among which the changes in lipid metabolism- and respiratory chain-related proteins were the most significant (Schauer et al., 2024).

In the pathological process of HFpEF associated with diabetic cardiomyopathy, activation of the mammalian target of rapamycin (mTOR) pathway and inhibition of mitophagy are also observed. Some studies have found that SGLT2i can mildly inhibit mTOR, promote mitophagy, clear dysfunctional mitochondria, and preserve the respiratory chain function of healthy mitochondria (Saha et al., 2023; Park et al., 2020).

SGLT2 inhibitors can inhibit inflammation and oxidative stress through multiple mechanisms, exerting positive effects on HF. They can improve myocardial injury and cardiac function, reduce the risk of hospitalization and cardiovascular death in HF patients, mainly reflected in the inhibition of inflammatory responses and alleviation of oxidative stress (Ijaz et al., 2024).

For example, dapagliflozin can reduce the expression of NLRP3 inflammasome in doxorubicin-induced HFpEF, thereby significantly decreasing the systemic expression levels of IL-1β, IL-6, TNF-α, and granulocyte colony-stimulating factor (G-CSF) in cardiomyocytes (Quagliariello et al., 2024). In a high-fat diet-induced HFpEF model, dapagliflozin significantly reduces cardiac macrophage infiltration and inhibits the expression of pro-inflammatory cytokines (IL-1β, TNF-α, CXCL10) (Crespo-Masip et al., 2025).

In terms of improving oxidative stress, SGLT2 inhibitors can potently block oxidative stress responses, inhibit cardiomyocyte hypertrophy and fibrosis, and reverse ventricular remodeling (Li et al., 2024b). Taking empagliflozin as an example, results from a randomized, double-blind, placebo-controlled study showed that empagliflozin significantly enhances antioxidant capacity by increasing the activities of serum SOD and glutathione peroxidase (GPx) (Eshraghi et al., 2025). Similarly, dapagliflozin can also reduce NADPH oxidase activity in myocardial tissue, decrease ROS production, and improve glutathione antioxidant reserves (Evans et al., 2022).

SGLT2 inhibitors (SGLT2i) also show great potential in improving myocardial fibrosis. Several indirect factors, such as ameliorating inflammation, alleviating cellular oxidative stress, and regulating calcium homeostasis in cardiomyocytes, can effectively reduce myocardial fibrosis. In addition, SGLT2i can directly act on fibroblasts. Studies have demonstrated that SGLT2i can significantly attenuate fibroblast activation induced by the TGF-β1/Smad3 pathway and reduce extracellular collagen deposition (Daud et al., 2021). Taking dapagliflozin as an example, after 8 weeks of intervention with dapagliflozin, glucose metabolism parameters (including blood glucose, glucose tolerance, and insulin sensitivity) in ZDF rats were significantly improved. Dapagliflozin also significantly reduced ventricular hypertrophy and restored diastolic function in ZDF rats (Feng et al., 2023).

#### 3.1.2 Current status of SGLT2 inhibitors in clinical treatment of HFpEF

SGLT2 inhibitors have been widely recognized in the clinical treatment of heart failure with preserved ejection fraction (HFpEF) and are an important therapeutic agent recommended by guidelines, with clear efficacy in reducing the risk of cardiovascular death or heart failure hospitalization (Anker et al., 2021).

The 2024 Chinese Guidelines for the Diagnosis and Treatment of Heart Failure recommend SGLT2 inhibitors for HFpEF patients to reduce the risk of the composite endpoint of heart failure hospitalization or cardiovascular death, as a Class I recommendation (Zhang, 2025). The 2023 Update of the ESC Guidelines for the Diagnosis and Treatment of Acute and Chronic Heart Failure also includes SGLT2 inhibitors for HFpEF treatment as a Class IA recommendation (SEC Working Group for the 2023 update of the 2021 ESC guidelines for the diagnosis and treatment of ac ute and chronic heart failure and SEC Guidelines Committee, 2024).

Pooled analyses show that SGLT2 inhibitors reduce the risk of cardiovascular death or heart failure hospitalization by 21% in HFpEF patients, with a 29% reduction in heart failure hospitalization. In terms of hemodynamic improvement, there is a significant decrease in the E/e' ratio, which is positively correlated with the recovery of mitochondrial function (Anker et al., 2021; Böhm et al., 2022). It indicates that SGLT2 inhibitors have shown certain potential for the treatment of HFpEF in clinical practice.

Furthermore, SGLT2 inhibitors have advantages in the co-management of diabetes, heart, and kidney. For HFpEF patients with comorbid type 2 diabetes mellitus (T2DM) or chronic kidney disease (CKD), they can reduce the risk of cardiovascular death or heart failure hospitalization while improving blood glucose and renal function. No titration is required, and tolerability is good, providing consistent benefits to patients (Jhund et al., 2022; Damman et al., 2014).

### 3.2 GLP-1 receptor agonists

Glucagon-like peptide-1 receptor agonists (GLP-1RAs) were initially used for the treatment of type 2 diabetes mellitus. As of 2024, GLP-1RAs have not yet been applied as therapeutic drugs for HFpEF in clinical practice (Ying et al., 2015). However, studies (Karlström et al., 2025) have shown that GLP-1RAs can directly activate glucagon-like peptide-1 receptors (GLP-1R) (Hullon et al., 2025) on mitochondria, and regulate mitochondrial biogenesis, optimize mitochondrial function, and counteract mitochondrial oxidative stress, thereby demonstrating potential therapeutic application value for HFpEF. For example, in a recent study (Shigeno et al., 2016), researchers found that the GLP-1 analog Exendin-4 upregulates AMPK activity, activates sirtuin 1 (SIRT1) deacetylase, promotes the expression of peroxisome proliferator-activated receptor gamma coactivator 1-alpha (PGC-1α), and reduces the production of mitochondrial ROS. GLP-1RAs also exert a positive effect on mitochondrial structure, researchers observed that in a HFpEF mouse model induced by a high-fat diet, liraglutide activates the Erbb4 receptor, inhibits the phosphorylation of protein kinase C alpha (PKCα) and extracellular signal-regulated kinase 1/2 (ERK1/2), reduces myocardial mitochondrial swelling and cristae fragmentation, and enhances adenosine ATP production simultaneously (Ni et al., 2024). As therapeutic drugs for diabetes, GLP-1RAs also have certain application potential in improving mitochondrial energy metabolism and optimizing the utilization of energy substrates. For instance, semaglutide promotes myocardial glucose oxidation via the Creb5/nuclear receptor subfamily 4 group A member 1 (NR4a1) axis. In HFpEF mice induced by transverse aortic constriction (TAC), semaglutide (Ma et al., 2024) downregulates the nuclear receptor NR4a1 and its translocation to mitochondria, promotes the entry of pyruvate into the tricarboxylic acid cycle, restores myocardial ATP levels to 85% of those in the normal group, and reduces mitochondrial lipid accumulation at the same time.

In addition to preclinical animal studies, findings from some clinical investigations have also indicated the potential application value of GLP-1RAs in HFpEF (Waqas et al., 2025). A real-world analysis of 1.67 million patients with HFpEF (Lee and Elmore, 2025) demonstrated that treatment with semaglutide and tirzepatide reduced the risk of heart failure hospitalization or all-cause death by 40%–58%, and this benefit was positively correlated with the degree of improvement in baseline mitochondrial function markers (such as serum mitochondrial DNA copy number). In patients with obesity-related HFpEF (BMI ≥30 kg/m^2^), after 48 weeks of treatment with semaglutide (2.4 mg/week), skeletal muscle mitochondrial density measured by magnetic resonance spectroscopy (MRS) increased by 18%, which was significantly associated with improvements in 6-min walk distance and left ventricular E/e' ratio (Butler et al., 2023).

### 3.3 FGF21 Analogues and signal regulation

**FIGURE 5:**
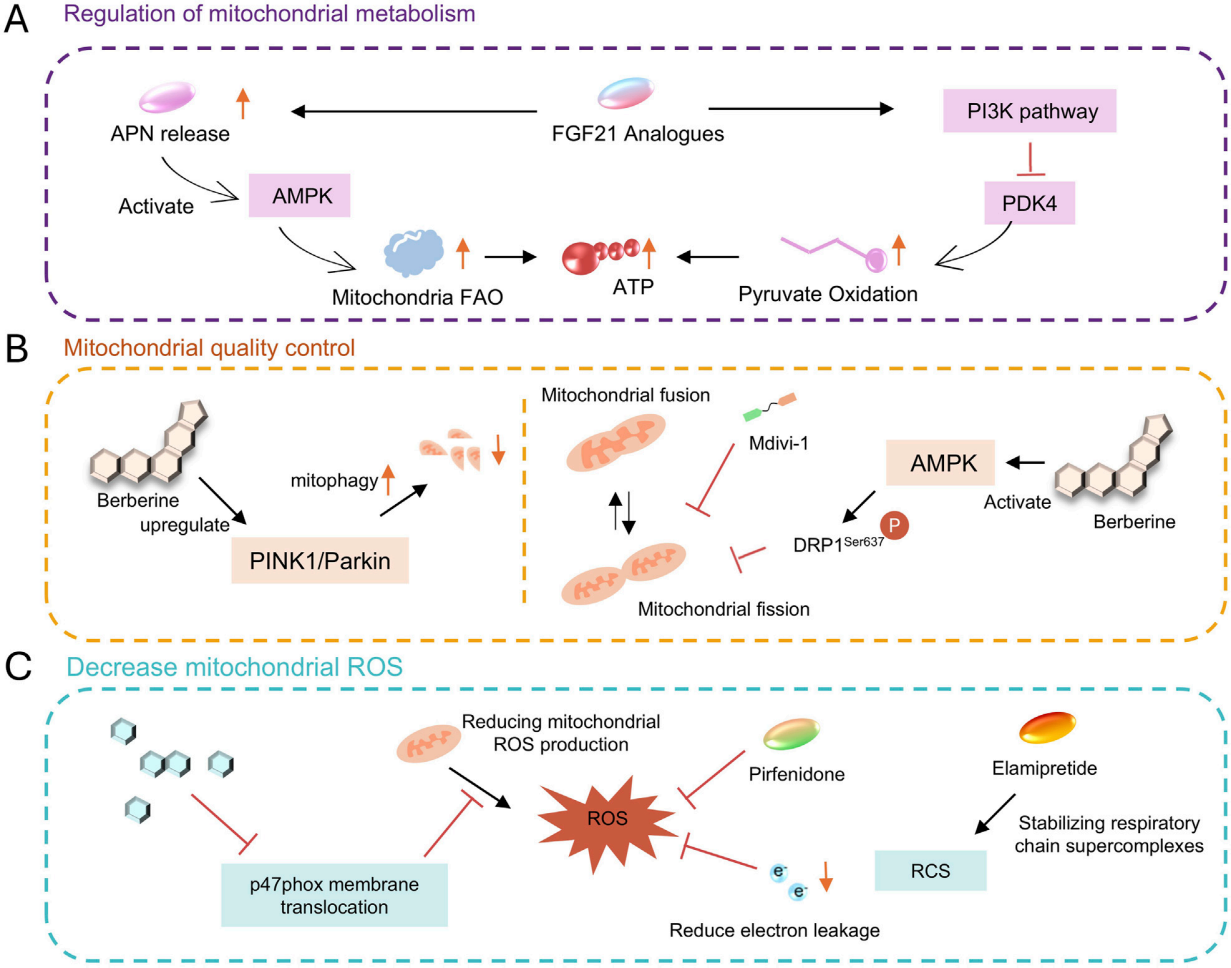
**(A)** metabolism regulation via AMPK, FGF21, etc. **(B)** quality control with mitophagy, fusion/fission modulation. **(C)** ROS reduction using various compounds.

Fibroblast growth factor 21 (FGF21) is a metabolic regulatory hormone, and recent studies have revealed its protective effect on mitochondrial function in HFpEF (Figure 5A). In uremic heart failure rat models, obese HF rat models, and hypertensive HF mouse models, FGF21 has been shown to improve mitochondrial function, thereby regulating cardiac remodeling and ameliorating HF (Li et al., 2019; Rupérez et al., 2018; Zeng et al., 2020).

A study demonstrated that FGF21 deficiency significantly exacerbates cardiac diastolic dysfunction in mice after HFpEF, while adipose-specific overexpression of FGF21 can significantly improve this condition. After injecting FGF21 adeno-associated virus (AAV-FGF21) into HFpEF mice, indices of cardiac diastolic function such as the E/A ratio increased, the E/E′ ratio decreased, pulmonary congestion and exercise intolerance were alleviated, and cardiac hypertrophy, oxidative stress, and fibrosis were also attenuated (Zhang K. et al., 2025).The cardioprotective effect of FGF21 against HFpEF is closely associated with its regulation of pyruvate oxidation and improvement of mitochondrial biological function (Zhang K. et al., 2025). In FGF21 gene-deficient HFpEF mice, cardiac mitochondrial pyruvate oxidation is impaired, and energy production by the respiratory chain is significantly reduced, however, FGF21 overexpression can significantly ameliorate this situation. *In vitro* cellular experiments have confirmed that FGF21 inhibits PDK4 by activating the PI3K pathway, thereby promoting pyruvate oxidation and improving mitochondrial energy-producing function, ultimately exerting a protective effect on cardiomyocytes (Zhang K. et al., 2025). In addition, FGF21 exerts cardioprotection by synergizing with adiponectin (APN). FGF21 can stimulate adipose tissue to release APN, which then activates the AMPK pathway via the AdipoR1 receptor, further inhibiting PDK4 and promoting fatty acid oxidation. Studies have shown that the protective effect of FGF21 on HFpEF is significantly attenuated in Apn knockout mice, confirming that APN is its key downstream mediator (Su et al., 2024).

Currently, recombinant FGF21 analogs (such as Pegbelfermin) have completed Phase II trials for non-alcoholic fatty liver disease, and clinical studies on their use in HFpEF are underway.

### 3.4 Natural compounds and active ingredients of traditional Chinese medicine

#### 3.4.1 Berberine

Berberine is an isoquinoline alkaloid extracted from plants such as Coptis chinensis (Figure 5B) (Gökçay Canpolat and Şahin, 2021). Numerous studies (Müller et al., 2025; Abudureyimu et al., 2024; Bode et al., 2021; Kitzman et al., 2024) have confirmed that it improves HFpEF by regulating mitochondrial dynamics. Berberine activates AMPK, which phosphorylates and inhibits dynamin-related protein 1 (Drp1) at Ser637, reducing its translocation to mitochondria and alleviating mitochondrial fragmentation. In HFpEF mouse models, Berberine reduces mitochondrial fission by 35% and improves the integrity of the mitochondrial network (Abudureyimu et al., 2023). Beyond regulating mitochondrial fission, berberine also protects cardiac function by upregulating PINK1/Parkin-mediated mitophagy in HFpEF, indicating its key role in HFpEF (Abudureyimu et al., 2020). Additionally, berberine exerts a certain effect in reducing cardiomyocyte apoptosis. Studies have shown that berberine decreases the rate of cardiomyocyte apoptosis by upregulating apoptosis-related proteins (such as BCL-2 and Bax) and downregulating the expression of Caspase-3, it also reduces endoplasmic reticulum stress in cardiomyocytes (Liao et al., 2018). Berberine also contributes to regulating calcium homeostasis. It improves sarcoplasmic reticulum calcium reuptake and shortens the diastolic calcium transient duration of cardiomyocytes by increasing SERCA2a activity and inhibiting excessive phosphorylation of RyR2. Meanwhile, by protecting the function of the mitochondrial calcium uniporter (MCU) complex, it enhances mitochondrial calcium buffering capacity, indirectly supporting energy synthesis (Abudureyimu et al., 2023).

#### 3.4.2 Icariin

Icariin, the main active ingredient of the traditional Chinese medicine Epimedium, exerts cardioprotective effects closely related to the activation of the NO-cGMP-PKG pathway (Figure 5C). Icariin increases myocardial nitric oxide (NO) production by regulating the expression and activity of endothelial nitric oxide synthase (eNOS) (Zeng et al., 2022). NO activates soluble guanylate cyclase (sGC), elevates cyclic guanosine monophosphate (cGMP) levels, and thereby activates protein kinase G (PKG). PKG reduces myofilament calcium sensitivity by phosphorylating cardiac myosin-binding protein C (cMyBP-C) and cardiac troponin I (cTnI), thereby improving myocardial relaxation (Song et al., 2008).

In the DOCA-salt-induced HFpEF rat model, after 6 weeks of treatment with icariin (10 mg/kg), myocardial ROS levels decreased by 45%, and mitochondrial function was partially restored. The expression of TNF-α and IL-6 was reduced by 38% and 42%, respectively (Scicchitano et al., 2021). The underlying mechanism involves inhibiting the membrane translocation of the NADPH oxidase subunit p47phox, thus blocking the positive feedback loop of ROS generation (Jia et al., 2023).

#### 3.4.3 Astragaloside

Astragaloside IV is the main active ingredient of the traditional Chinese medicine Astragalus membranaceus, and its cardiovascular protective effects have been confirmed in long-term studies (Zang et al., 2020). Moreover, astragaloside IV is beneficial for improving oxidative stress. Recent studies have shown that astragaloside IV can effectively inhibit the expression of inflammatory factors in myocardial tissue and improve abnormal energy metabolism in HFpEF rats, with significant reductions in the expression levels of TNF-α and IL-6. A recent study by Xiao Wang et al. found that in the Dahl-ss rat HFpEF model, the high-dose astragaloside IV group (40 mg/kg) significantly reduced serum NT-ProBNP levels, while downregulating the protein expression of vWF, TNF-α, and IL-6. Additionally, astragaloside IV can ameliorate the oxidative stress state of rat cardiomyocytes, promote the expression of VEGF and CD31, enhance angiogenesis and endothelial function, increase LVEF in HFpEF rats, and improve cardiac diastolic function (Wang X. et al., 2024).

### 3.5 Targeted therapy for ROS and oxidative stress

Antioxidant therapy, including supplementation with antioxidants such as coenzyme Q10 (Samuel et al., 2022) and vitamin E (Ratchford et al., 2019), can improve mitochondrial dysfunction and energy metabolism abnormalities caused by ROS accumulation, thereby alleviating HFpEF symptoms (Sobirin et al., 2019). Additionally, activating the endogenous antioxidant system, for example, high-dose icariin can upregulate SOD2 expression and reduce myocardial oxidative stress in HFpEF rats is also beneficial for improving HFpEF symptoms (Russomanno et al., 2017). The nuclear factor erythroid 2-related factor 2 (Nrf2) signaling pathway, a key regulator of antioxidant defense, is often inhibited in HFpEF, further weakening cellular oxidative stress response capacity (Liu et al., 2018). Multiple studies have shown that activating the Nrf2/SLC7A11/GPX4 axis can increase endogenous glutathione (GSH) levels and enhance the antioxidant capacity of cardiomyocytes, thereby reducing ROS accumulation (Liu et al., 2018; Chen et al., 2022; Wei et al., 2025; Han et al., 2024).

### 3.6 Mitochondrial inheritance and genetic changes

The genetic basis of mitochondrial dysfunction in HFpEF offers a novel perspective for understanding its pathogenesis. Clinical studies have revealed significant interindividual heterogeneity among patients, suggesting that genetic factors may drive the heterogeneity of mitochondrial phenotypes. Regarding nuclear gene variations, the loss of the mitochondrial binding domain in hexokinase 1 (HK1) (Tatekoshi et al., 2025) leads to its dissociation from mitochondria, which inhibits angiogenesis via enhanced O-GlcNAcylation. The ΔE1HK1 mouse model spontaneously progresses to HFpEF, validating the pathogenicity of this genetic defect (Mishra and Kass, 2021). At the mitochondrial genome level, loss-of-function mutations in nicotinamide nucleotide transhydrogenase (NNT) exhibit a protective effect (Pepin et al., 2025). Nnt^−/−^ mice did not develop diastolic dysfunction under HFpEF induction (Pepin et al., 2025). The mechanism is associated with the maintenance of NAD^+^ levels and the mitigation of oxidative stress, supporting the potential of NAMs (nicotinamide adenine dinucleotide-based therapeutics) to improve mitochondrial function by modulating NAD^+^ metabolism (Wang Y. C. et al., 2024).

Systems biology studies have further revealed genetic regulatory networks. The ZSF1-obese rat model demonstrates that genes related to mitochondrial structure (e.g., MFN2) and oxidative metabolism pathways are significantly downregulated in the HFpEF heart, accompanied by reduced ATP production and calcium handling defects (Doiron et al., 2025). These findings are consistent with clinical data showing a decrease in MFN2 expression in the skeletal muscle of human HFpEF patients, suggesting that nuclear-mitochondrial crosstalk dysregulation may be a shared mechanism (Alves et al., 2024). Although genome-wide association study (GWAS) research has not yet fully identified mitochondrial genetic loci for human HFpEF, existing evidence indicates that genetic variations in genes such as HK1 and NNT contribute to disease pathogenesis by affecting mitochondrial-cytoplasmic metabolic coupling and redox balance, thereby providing targets for precision interventions based on genetic background (Tatekoshi et al., 2025).

### 3.7 Other targeting strategies and investigational drugs

Pirfenidone, an approved drug for idiopathic pulmonary fibrosis, has shown translational potential in HFpEF (Lewis et al., 2021), as it improves mitochondrial function through three mechanisms: it inhibits the TGF-β/Smad signaling pathway, thereby reducing fibroblast activation and decreasing collagen I/III deposition by 42%, it scavenges mitochondrial ROS, leading to a 67% reduction in NOX4 activity, and it blocks the IL-6/JAK2/STAT3 pathway, resulting in a 53% lowering of MCP-1 expression. The preserve-HF trial showed that after 24 weeks of treatment with pirfenidone (2403 mg/d), the left ventricular stiffness constant β in HFpEF patients significantly decreased. Currently, the Phase III FIBRO-HF trial (NCT05892341) of low-dose pirfenidone (1200 mg/d) in combination with sacubitril/valsartan is underway (Aimo et al., 2022).

Drp1 inhibitors (e.g., Mdivi-1): Mdivi-1 blocks its oligomerization and mitochondrial fission by inhibiting Drp1 GTPase activity. In diabetic cardiomyopathy models, Mdivi-1 improves mitochondrial morphology and function and alleviates myocardial fibrosis. Although it has not yet entered clinical trials for HFpEF, the Drp1-inhibiting effect of berberine provides a theoretical basis for its application (Guan et al., 2023).

Mitochondrial antioxidants (e.g., Elamipretide): Elamipretide stabilizes respiratory chain supercomplexes by binding to cardiolipin, reducing electron leakage and ROS production. In heart failure with reduced ejection fraction (HFrEF), it improves myocardial energy metabolism, and research on its effect in HFpEF is ongoing (Hattori et al., 2024; Zhang et al., 2023).

## 4 Summary and future direction

Although mitochondria-targeted therapy has brought new hope for heart failure with preserved ejection fraction (HFpEF), it still faces multiple challenges: In terms of disease heterogeneity, HFpEF encompasses various phenotypes such as obese, hypertensive, and aging-related subtypes, with distinct dominant mechanisms of mitochondrial dysfunction among different subtypes, necessitating personalized intervention strategies, Regarding model limitations, existing animal models (e.g., ZSF1 rats, HFD + L-NAME mice) cannot fully replicate the metabolic-inflammatory crosstalk characteristics of human HFpEF, which impairs the predictive value for clinical translation, There is a lack of biomarkers, and there is an urgent need to develop circulating biomarkers reflecting myocardial mitochondrial function (such as cell-free mitochondrial DNA [mtDNA], cardiolipin derivatives) for efficacy monitoring, In terms of drug delivery, technical breakthroughs are still required to achieve targeted delivery to myocardial mitochondria, enhance local drug concentration, and reduce off-target effects.

Future research can leverage multi-omics technologies and advanced methodologies to deeply explore mitochondrial mechanisms and develop precise interventions, including integrated spatial multi-omics analysis—combining single-cell transcriptomics (e.g., scRNA-seq), spatial transcriptomics (10x Visium HD), and metabolomics to map the mitochondrial functional atlas of cardiac microregions in HFpEF and decipher mitochondrial interaction networks among different cell types (cardiomyocytes, macrophages, endothelial cells), gene editing and cell therapy—using adeno-associated virus (AAV) vectors to deliver mitochondrial protective genes (e.g., FGF21, SOD2) or achieving precise regulation through CRISPR-Cas9-mediated editing of Drp1 loci, while exploring the potential of mitochondrial transplantation or mitochondria-enhanced mesenchymal stem cell therapy in HFpEF, and artificial intelligence-driven drug design—screening novel allosteric inhibitors using deep learning algorithms based on the HFpEF mitochondrial protein structure library (e.g., PDK4, Drp1), with network pharmacology enabling systematic analysis of multi-target mechanisms of traditional Chinese medicine ingredients such as icariin.

To advance mitochondria-targeted drugs toward clinical application, the following pathways should be followed: On the basis of precise phenotyping of HFpEF, screen potential beneficiary populations using biomarkers (e.g., plasma FGF21, PDK4 activity), adopt adaptive clinical trial designs (e.g., platform trials) to simultaneously evaluate multiple mitochondria-targeted drugs and improve research and development efficiency, comprehensively assess therapeutic benefits by combining patient-reported outcomes (e.g., KCCQ scores) with traditional cardiac function indices (E/e', exercise tolerance), and pay attention to long-term safety, particularly the potential neuromuscular side effects of respiratory chain inhibitors.
